# Why Be a Shrub? A Basic Model and Hypotheses for the Adaptive Values of a Common Growth Form

**DOI:** 10.3389/fpls.2016.01095

**Published:** 2016-07-26

**Authors:** Frank Götmark, Elin Götmark, Anna M. Jensen

**Affiliations:** ^1^Department of Biological and Environmental Sciences, University of GothenburgGöteborg, Sweden; ^2^Mathematical Sciences, Chalmers University of Technology and University of GothenburgGöteborg, Sweden; ^3^Department of Forestry and Wood Technology, Linnaeus UniversityVäxjö, Sweden

**Keywords:** woody plants, stem, multi-stemmed, shrubland, scrub, tree, growth, canopy

## Abstract

Shrubs are multi-stemmed short woody plants, more widespread than trees, important in many ecosystems, neglected in ecology compared to herbs and trees, but currently in focus due to their global expansion. We present a novel model based on scaling relationships and four hypotheses to explain the adaptive significance of shrubs, including a review of the literature with a test of one hypothesis. Our model describes advantages for a small shrub compared to a small tree with the same above-ground woody volume, based on larger cross-sectional stem area, larger area of photosynthetic tissue in bark and stem, larger vascular cambium area, larger epidermis (bark) area, and larger area for sprouting, and faster production of twigs and canopy. These components form our Hypothesis 1 that predicts higher growth rate for a small shrub than a small tree. This prediction was supported by available relevant empirical studies (14 publications). Further, a shrub will produce seeds faster than a tree (Hypothesis 2), multiple stems in shrubs insure future survival and growth if one or more stems die (Hypothesis 3), and three structural traits of short shrub stems improve survival compared to tall tree stems (Hypothesis 4)—all hypotheses have some empirical support. Multi-stemmed trees may be distinguished from shrubs by more upright stems, reducing bending moment. Improved understanding of shrubs can clarify their recent expansion on savannas, grasslands, and alpine heaths. More experiments and other empirical studies, followed by more elaborate models, are needed to understand why the shrub growth form is successful in many habitats.

“…since Theophrastus (born c. 370 BC), botanists have generally distinguished between trees, shrubs, and herbs.” (Petit and Hampe, 2006, p. 189)

“Shrubiness is such a remarkable adaptive design that one may wonder why more plants have not adopted it.” (Stutz, 1989, p. 325)

## Introduction

Trees and shrubs are two major growth forms in many natural and semi-natural habitats. Here, we focus on shrubs, a widespread category of woody plants, and elucidate their adaptive significance. We present a model based on scaling relationships where shrubs are compared with trees, outline hypotheses for the adaptiveness of shrubs, and test one of the hypotheses, based on the literature.

Many theoretical and empirical studies of trees address their adaptive significance, for instance variability in height among species, and maximum height (e.g., Horn, 1971; Ryan and Yoder, 1997; Loehle, 2000). In contrast, the adaptive significance of shrubs is only discussed briefly in the literature. For instance, Whittaker and Woodwell (1968, p. 11) stated that shrubs “may have high production per unit leaf weight and surface…and smaller expenditure of this production on supporting stem and branch tissue than is the case in forest trees.” Givnish (1984, p. 78) suggested that shrubs are favored in open habitats where tree crowns have been destroyed, by having “more meristems active, [and] more potential points for stem regeneration.”

Another suggestion is that the shrub growth form is “a design strategy of relatively small, low-investment, low risk, “throwaway” stems that are expendable in high-stress environments” (Wilson, 1995, p. 92). Stutz (1989) stated that shrubs usually are tall enough to dominate herbs and do not need to rebuild as much biomass each year as herbs. On the other hand, shrubs often occur in grassland, for instance savanna, where grasses and/or fires may control woody vegetation, including shrubs (Bond and van Wilgen, 1996; Sholes and Archer, 1997). Shrubs are sometimes discussed on the basis of their low, broad canopy in disturbed habitats, and Givnish (1984) argued that such a canopy is favored by multiple stems. It is often suggested that shrubs are associated with disturbed and stressful environments (e.g., Rundel, 1991; Givnish, 1995; Sheffer et al., 2014). However, elaborate hypotheses and models for the adaptive significance of the shrub growth form seem to be lacking. Moreover, the recent expansion of shrubs in several regions globally (e.g., Naito and Cairns, 2011; Formica et al., 2014) motivates more basic research about shrubs.

Below, we first define “tree” and “shrub.” Because shrubs have been neglected compared to herbs and trees (see Discussion), we briefly outline their importance. We then describe our basic model and four hypotheses that potentially can explain the adaptive significance of shrubs, compared to trees. Our main contributions are the basic model (Section The Basic Model and Hypotheses), and Hypothesis 1 and the preliminary test of it (Section Hypothesis 1: The Multiple Stems of a Small Shrub Give Faster Growth than for a Small Tree). The Hypotheses 2, 3 and 4 (Sections Hypothesis 2: The Fast Maturity of Shrubs Enables Earlier Seed Production Compared to Trees, Hypothesis 3: The Multiple Stems in Shrubs Insure Future Survival and Growth if One or More Stems Die, and Hypothesis 4: The Short Stems of Shrubs Improve Survival through Three Traits, Compared to Tall Tree Stems) are complementary and also important ideas, supported by some evidence. Finally, we discuss ecological aspects of shrubs and trees, and identify research needs.

### Delimitation and definition

It is sometimes difficult to identify a woody plant as a tree or a shrub, and intermediate forms exist (see Rundel, 1991; Wilson, 1995). Sheffer et al. (2014) stated “In contrast to shrubs, trees have a single stem, but this distinction is not absolute…9.2% of the tree species we analyzed were also qualified as shrubs by some contributors in the trait database.” In tropical rainforest, the woody growth forms are diverse, with more forms than just tree/shrub (see Givnish, 1984, Table 4;Rundel, 1991). In South African savanna, in a study of 23 woody species, Zizka et al. (2014) recognized shrubs (mean value: 13 stems), SSTs (“shrubs sometimes small trees,” 3.6 stems) and trees (2.2 stems). Some shrubs are semi-woody, being woody in the lower stem parts and herbaceous in the upper (trees also have herb-like shoots that become woody with time).

**Figure 1:**
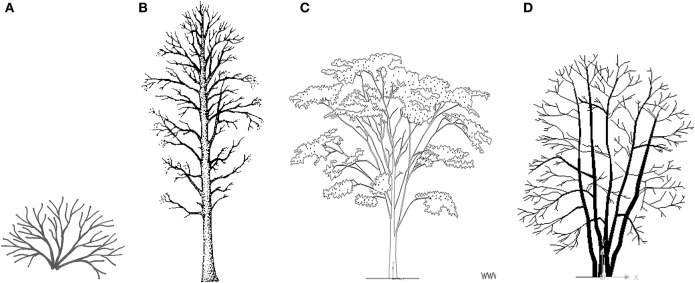
**Four types of woody plants: (A) Shrub, here with five stems, branching as in the basic model (about 50 cm tall). (B)** Tree with main stem throughout the plant. **(C)** Tree with short main stem with many branches, forming most of the plant. **(D)** Tree with multiple stems. **(C,D)** are from Ceco.NET, **(B)** is from Natural Resources Canada (red alder; tidcf.nrcan.gc.ca), and **(A)** is our own drawing.

**Figure 2:**
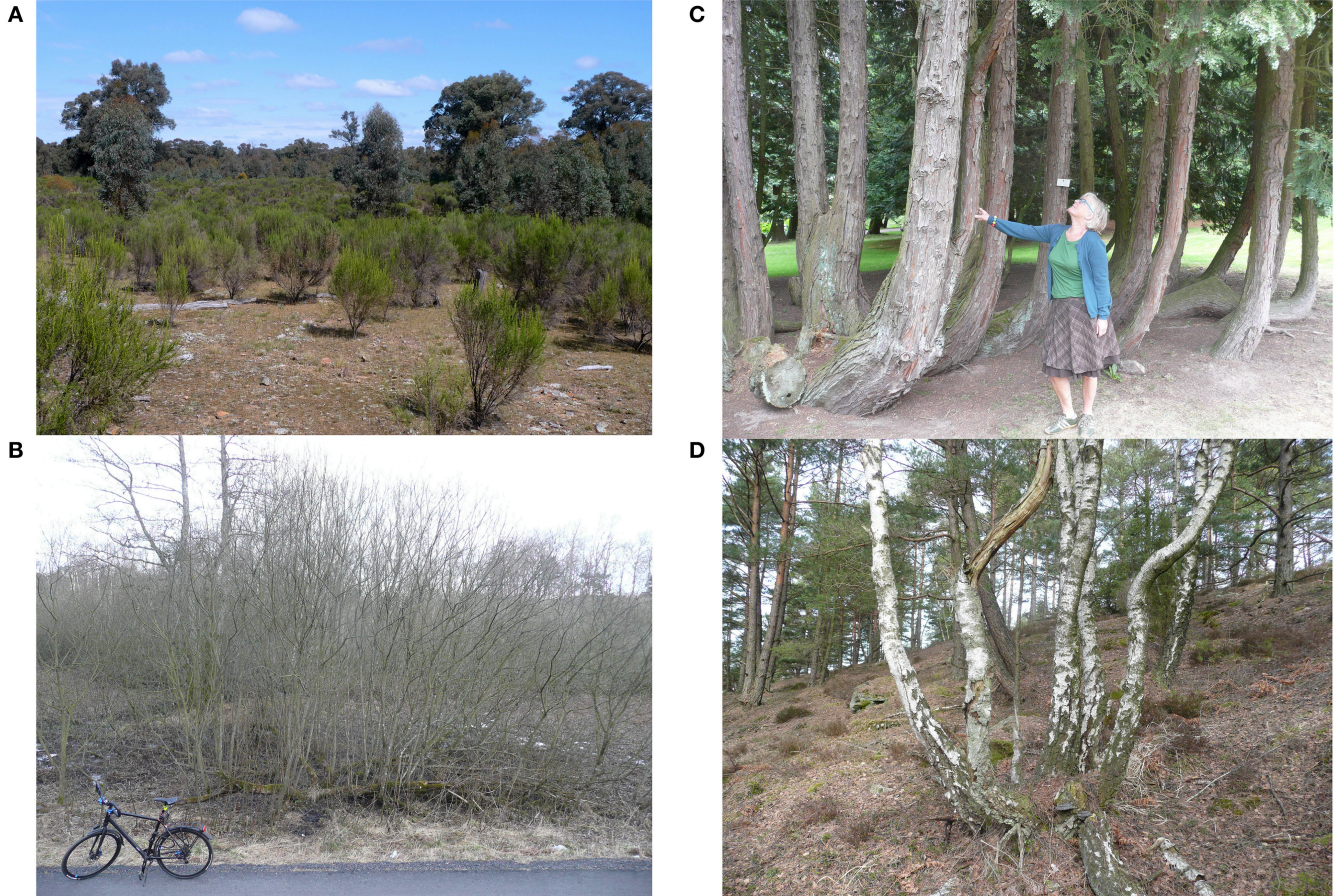
**Two species of shrubs and two species of trees, multi-stemmed: (A) *Cassinia arcuata* (Asteraceae), Drooping Cassinia or Chinese Scrub, an evergreen shrub in central Victoria, Australia**. This species has colonized thousands of hectares in the area during the last 40 years, when land use changed (see Lunt, 2011). The trees are *Eucalyptus sideroxylon* (Red Ironbark). **(B)** A large *Salix* sp. shrub (probably a hybrid) in winter on moist ground in Sweden, with horizontal growth by sprouts on lying stems. Deciduous *Salix* spp. are common especially on moist soils in cold and temperate regions in the northern hemisphere. **(C)** *Chamaecyparis lawsoniana* (Cupressaceae) or Port-Orford-cedar, an evergreen conifer from western North America. It is normally single-stemmed but may become multi-stemmed after damages, e.g., from browsing (picture from botanical garden, Sweden). **(D)** *Betula pendula* (Betulaceae), Silver Birch or Warty Birch in multi-stemmed version probably caused by browsing or cutting damage on seedling/sapling (Pixbo, SW Sweden). Note self-thinning (dead stems). Normal single-stem birches grow in the background. Note also uprising stems of the multi-stemmed trees in **(C,D)**, which would reduce the bending moment of heavy leaning stems (see Discussion and Figure 4). Photographs: Ian Lunt **(A)** and Frank Götmark **(B–D)**.

Here, we define a tree as a tall perennial plant with a single self-supporting woody stem, and a shrub as a short perennial plant with multiple self-supporting woody stems, branching at or near the ground. However, trees can have multiple stems, and the shrubs we discuss below range from very small (e.g., *Vaccinium* spp., about 0.2 m tall) to large *Corylus* spp. (up to about 10 m tall). Figure 1 illustrates a shrub of a common type (about 50 cm tall); a tree with one central stem (many conifers and angiosperms); a tree with one short stem, branching early to produce a broad canopy forming >50% of the height of the tree (e.g., *Ulmus* spp., savanna trees); and a tree with multiple stems which we suggest may be distinguished by stem form as well as height (see Discussion). Photographs in Figure 2 illustrate two types of shrubs and two multi-stemmed trees. Shrub-like bamboos are also relevant, some of which have strong stems more than 25 m tall (Wang et al., 2014) and may also dominate trees (Griscom and Ashton, 2003), but we did not include them in our literature review below.

Despite problems in defining some species as shrubs, the term shrub is widely used and shrubs are important in many ecosystems (see next section). In addition, biology and ecology contain many terms that are difficult to define precisely (e.g., “forest”) but useful in research and management.

### The occurrence and ecological importance of shrubs

Shrubs are important components in at least 9 of 11 global biomes (Archibold, 1995; see also McKell, 1989), forming much of the vegetation in tropical savannas, arid regions, Mediterranean ecosystems, and polar and high mountain tundras. They are also frequent in terrestrial wetlands and in the understory and canopy gaps in forests, where both shade-tolerant and pioneer (shade-intolerant) shrubs occur (e.g., Denslow et al., 1990).

Olson et al. (2001) classified 14 terrestrial biomes, and “shrubland” or “scrub” occur in the name of 5 biomes. Shrubs occur in at least 13 of the 14 biomes. Gong et al. (2013) used satellite data to estimate global land-cover types; forest covered 28.4% of the land and shrubland covered 11.5%. Because shrubs also occur in forest, they grow, or can grow, on about 40% of the land surface. Shrubland was defined as having a vegetation cover of >15%, but some bare land with sparse vegetation also contains shrubs (see Gong et al., 2013), so the total area where they can grow might be close to 45% of the global land surface.

Given the vast global distribution of shrubs, they are important for climate control, soil stabilization and production, ecosystem water balance, carbon uptake and storage, and for many associated species such as grazing and browsing mammals and livestock, birds, fungi, and invertebrates. “Nurse plants” favor other plants, including trees, and in a review of such plants “shrubs were the dominant nurse life-form” (Filazzola and Lortie, 2014). Moreover, shrubs exhibit high species richness in several regions on the earth (Qian, 2015; Qian and Ricklefs, 2015; see also Rundel, 1991). Currently, shrubs and “shrubification” are much studied in tropical and temperate grassland and in arctic and other cold habitats that lack trees, often in relation to climate change (e.g., Hallinger et al., 2010; Ratajczak et al., 2012; Formica et al., 2014; Ogden, 2015).

The next section describes our basic model, which is relevant for Hypothesis 1 in Section Hypothesis 1: The Multiple Stems of a Small Shrub Give Faster Growth than for a Small Tree. All our four hypotheses focus on the adaptive value of shrubs compared with trees. For trees, we assume that their main adaptive value or advantage is height development, leading to elevated canopies that shade competitors (including shrubs) and large root systems that also help dominate shrubs. In addition, a tall tree with a large canopy can potentially produce more seeds and disperse pollen and seeds more widely.

## The basic model and hypotheses

**Figure 3:**
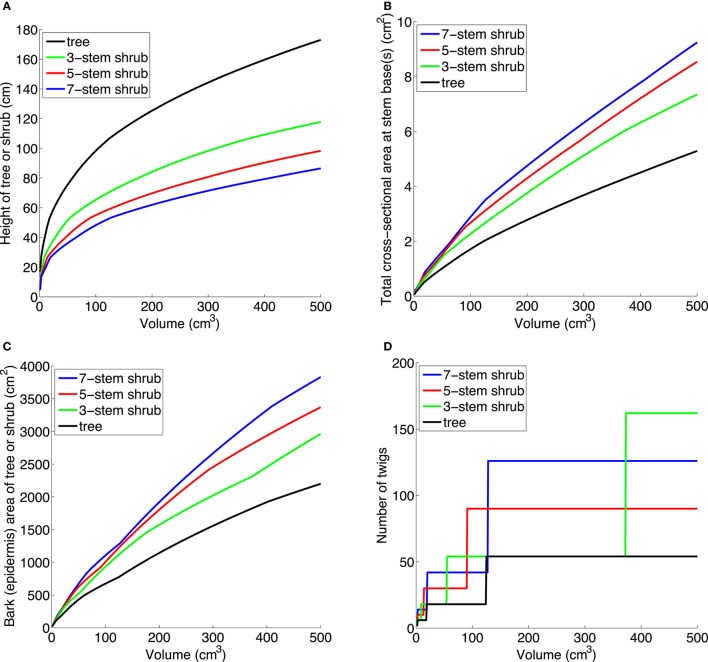
**(A)** Trees are taller than shrubs with the same above-ground woody volume. **(B)** A small shrub with the same above-ground woody volume as a small tree has a larger total cross-sectional area at stem base(s), increasing with number of stems. **(C)** A small shrub with the same above-ground woody volume as a small tree has a larger surface area, increasing with number of stems. This is true for bark (epidermis) as shown here, but also for sprouting area, cambium area, and area of photosynthetic tissue on and within stem. **(D)** A small shrub with the same above-ground woody volume as a small tree produces twigs (outermost generation of branches) faster than a tree. The parameter values in **(A–C)** are: *a*_*t*_ = *a*_*s*_ = 2, *p* = 0.5, *l*_*min*_ = 20, *r*_*tip*_ = 0.1, *b*_*t*_ = *b*_*s*_ = 0.0075, *g*_*s*_ = 1.

To explore functional trait differences between single- and multi-stemmed woody plants (trees vs. shrubs), we built a basic volume-based growth model. Biomass partitioning occurs only between above-ground woody parts, thus foliage and roots are not included in the model. The following traits were studied: cross-sectional stem area, bark surface area, branching, canopy development (branching), and stem bending moment (intuitively, the strain when forces act on the stem so that it bends). We modeled above-ground woody biomass [that is, stem(s) and branches] using the functions *V*_*t*_(*h*_*t*_) and *V*_*s*_(*h*_*s*_,*n*), which give the volumes of a tree of height *h*_*t*_, and a shrub of height *h*_*s*_, and number of stems *n*. For a given volume *v* we can solve *V*_*t*_(*x*) = *v* and *V*_*s*_(*x*,*n*) = *v* numerically and obtain heights *h*_*t*_(*v*) and *h*_*s*_(*v*,*n*), compared in Figure 3A.

For simplicity, tree and shrub volumes *V*_*t*_(*h*_*t*_*)* and *V*_*s*_(*h*_*s*_,*n*) are calculated by modeling stems and branches as truncated cones with basal radius proportional to length, or as cylinders when the basal radius is small enough. We explain the parameters in Table 1; all are constants which can be varied freely. When a stem reaches the length *l*_*min*_, branches of length p^*^l_*min*_ are added, which then grow proportionally in length with the main stem. We add *a*_*t*_ branches per stem for a tree and *a*_*s*_ for a shrub, corresponding to Whitney (1976) branching coefficients *a*_*t*_+1 and *a*_*s*_+1, respectively (Whitney counts the stem tip as a child branch; we do not). These branches in turn get “child branches” in the same way when they grow long enough. The model does not include thinning within individuals during growth, so we only apply it to small trees and shrubs.

**Table 1:** **Definition of parameters used in the basic model**.

**Parameter**	**Definition**
*n*	The number of stems.
*a_*t*_* and *a_*s*_*	The number of child branches added in each step to each parent branch/stem for trees and shrubs, respectively.
*p*	The ratio of the length of a child branch and the length of its parent branch/stem.
*l*_*min*_	The length at which a branch or stem gets child branches (in cm).
*r*_*tip*_	The radius of the outermost tip of a branch or stem (in cm).
*b_*t*_* and *b_*s*_*	The ratio of the basal radius of a stem or branch and its length *h* for trees and shrubs, respectively (an exception is made when *b_t_*·*h* or *b_s_*·*h* is less than *r*_*tip*_; the basal radius is then set to *r*_*tip*_, making the stem or branch a cylinder).
*g_*s*_*	The ratio of the growth rate of the above-ground woody volume of a shrub and that of a tree (we set *g_*s*_* = 1, but in case of e.g., known faster growth rate in a shrub, it could be changed).

Note that the above description is simplified: to avoid child branches p^*^l_*min*_ cm long appearing out of nowhere when parent branches reach the length *l*_*min*_ cm (making the volume functions discontinuous), child branches begin to grow when parent branches are 2/3^*^l_*min*_ cm long, and grow linearly to reach the length p^*^l_*min*_ cm when the parent branch is *l*_*min*_ cm long. The number 2/3 is rather arbitrary, but affects the results very little—it only specifies how the discontinuous parts of the function are “glued together.” The parameters *r*_*tip*_ (the radius of a branch tip), *b*_*t*_, and *b*_*s*_ (the ratio of the basal radius of a stem or branch and its length for trees and shrubs, respectively), *l*_*min*_, and *p* were chosen from inspection of small trees and shrubs of several species, to be: *a*_*t*_ = *a*_*s*_ = 2, *p* = 0.5, *l*_*min*_ = 20 cm, *r*_*tip*_ = 0.1 cm, *b*_*t*_ = *b*_*s*_ = 0.0075, *g*_*s*_ = 1. All parameters probably vary among species and habitats, but we have tried different realistic values and the scaling relationships between trees and shrubs seen in Figure 3 still hold. Note that to reach the same height as a tree, a shrub with *n* stems and with *a*_*t*_ = *a*_*s*_, *b*_*t*_ = *b*_*s*_, must increase in above-ground woody volume *n* times as fast as a tree (that is, *g*_*s*_ = *n*). This follows since one shrub stem with its branches is modeled the same as a tree stem with branches.

Once we have *h*_*t*_(*v*) and *h*_*s*_(*v*,*n*) we can calculate other important traits, for example the basal radius of a stem and thus the total cross-sectional area at stem base(s). Investing in multiple stems, compared to a single stem, gives a greater total cross-sectional area at the stem base(s), increasing with *n* (Figure 3B). We can also calculate the total surface area of stem(s) and branches. Investing in multiple stems gives a greater total bark surface area, increasing with *n* (Figure 3C). The same holds for the stem-photosynthetic area, the area of vascular cambium, and area for sprouting, e.g., on the lower 25% of the stems (all graphs would be similar to Figure 3C). All these results are illustrations of the general mathematical principle that volume and area scale differently. The number of twigs (outermost generation of branches) is larger for shrubs than for trees, given the same above-ground woody volume (Figure 3D). A more realistic twig model requires knowledge of the relative thinning and allocation strategies of trees and shrubs.

Our model uses a simple proportional relationship between stem height and basal radius for small stems (Whittaker and Woodwell, 1968; Niklas, 1994). In Equation (5) in Niklas and Spatz (2004) a relationship L = k_5_D^2∕3^ − k_6_ is derived between height L, basal stem diameter D, and empirically determined constants k_5_ and k_6_. This relationship is a good model for both small and large trees (as opposed to the common model L = kD^2∕3^ for large trees). Substituting this relationship instead of the simple proportional function in our model, the functions *V*_*t*_(*h*_*t*_*)* and *V*_*s*_(*h*_*s*_,*n*) will change, but the height and area comparison between trees and shrubs will not be much affected (see graphs in Data Sheet 1 in Supplementary Material). That is, the graphs in Figure 3 would be similar.

**Figure 4:**
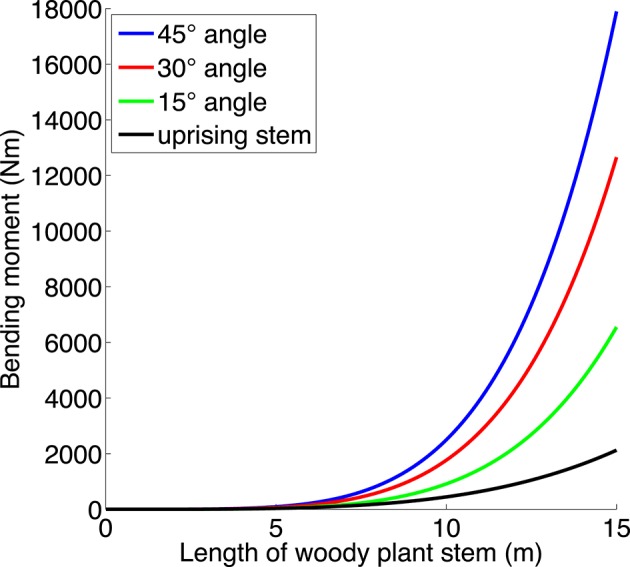
**The bending moment (Nm) as a function of the length of a straight stem (neglecting branches and foliage) growing in an angle of 15, 30, and 45⋅ from the vertical, and the bending moment for a more uprising stem such as a multi-stemmed tree often has (cf. Figures 1D, 2C,D)**. The latter stem first grows at an angle of 20⋅ off vertical, and when it has reached a horizontal distance of 1 m from its starting point, it grows straight upwards. Taper function from Niklas and Spatz (2004). (For calculations, see Supporting Information).

The bending moment around the origin of a point-mass at location **a** is |**F**|·|**b**|, where **F** is the force applied to the mass, and **b** is the component of **a** which is at right angles to **F**. We set the origin at the stem base. In our case the force will be gravity which acts vertically, so that the bending moment increases the farther we get from the origin horizontally. This is why a straight stem will have higher bending moment the more it leans outwards. Since a stem is not a point mass, we have to add all the contributions along its length, which leads to an integral. For simplicity, we omit the branches, and we use the stem taper function from Niklas and Spatz (2004; Figure 4; calculations in Data Sheet 1 in Supplementary Material). We use these results in Hypothesis 4.

All calculations are implemented in Matlab (see Supporting Information).

### Hypothesis 1: the multiple stems of a small shrub give faster growth than for a small tree

We suggest that a small shrub has at least six functional traits that lead to higher growth rate than in a small tree with the same above-ground woody volume and grown under similar conditions.

*First*, shrubs have greater sapwood area compared to trees of the same above-ground woody volume. When partitioning biomass to multiple stems instead of height growth (Figure 3A), shrubs develop a larger total stem cross-sectional area than in a tree (Figure 3B). Assuming similar heartwood proportions and sapwood efficiency in shrubs and trees, a larger total stem cross-sectional area would give a larger functional stem sapwood area. As hydraulic conductance scales positively with leaf area and mass (see Mencuccini, 2003; Rance et al., 2014), this indicates that a small multi-stemmed shrub may produce more leaves than a small tree with a single stem. While a greater sapwood area would result in greater rate of respiration (Lavigne and Ryan, 1997), we argue that this is negligible compared to the potential greater canopy C assimilation in shrubs. For plants of the same size, for example 500 cm^3^ volume, our model predicts a 39, 61, and 75% greater total cross-sectional sapwood area in shrubs with 3, 5, and 7 stems, compared to a single-stemmed tree (Figure 3B). Although slope (sapwood area to leaf area ratios) depends on species, growth conditions, and leaf water conductance (Whitehead et al., 1984; McDowell et al., 2002), recent work on different tropical trees suggests that cross-sectional sapwood area is a critical morphological trait for growth and biomass accumulation (van der Sande et al., 2015). Further, there is evidence that shrubs have higher canopy density than trees in savanna (total leaf area in m^2^ per canopy volume in m^3^; Zizka et al., 2014) and have higher leaf area index (LAI, m^2^ m^−2^) than trees in several mesic forests (Knapp et al., 2008: p. 620). Put differently, the larger sapwood area of a small shrub could potentially support a greater leaf area/mass than in a small tree, leading to higher C uptake and growth rate.

*Second*, shrubs have greater stem-photosynthetic area for additional C acquisition compared to trees. Although foliage in most shrubs and trees is the primary producer of photosynthates, C assimilation also occurs in stems and twigs (Stutz, 1989; Nilsen, 1995; Pfanz et al., 2002; Vick and Young, 2009; Avila et al., 2014). Our model predicts a 35, 53, and 74% greater total epidermis area in shrubs with 3, 5, and 7 stems, compared to a single-stemmed tree (for 500 cm^3^ above-ground woody volume, see Figure 3C). Reported light-saturated rates of bark photosynthesis (*A*_sat_) at ambient CO_2_ (360–400 ppm) are lower (0.6–2.2 μmol CO_2_ m^−2^ s^−1^) than foliage rates (7–35 μmol CO_2_ m^−2^ s^−1^; Wullschleger, 1993; Wittmann and Pfanz, 2007; Jensen et al., 2012, 2015), but this related stem area advantage for shrubs may be especially beneficial during leafless periods such as early spring and late autumn, after severe droughts (Maurits et al., 2015), or after insect defoliation events. However, the primary function of corticular photosynthesis may not be net photosynthetic uptake of CO_2_ but rather sustaining physiologically active tissues by re-assimilation of respired CO_2_ (Pfanz et al., 2002; Wittmann and Pfanz, 2007; Teskey et al., 2008). Multi-stemmed shrubs may thereby be able to maintain a greater cambium area than small single-stemmed trees.

*Third*, shrubs have greater total cambium area than trees with the same above-ground woody volume, facilitating greater secondary xylem and phloem growth in all emerging stems and branches. However, a greater active cambium area requires additional C investment both in cambium development and maintenance. A greater total cambium area *per se* does not ensure higher growth rates, but is a precondition for accelerated growth. So shrubs have an advantage over trees for plants grown without resource limitations.

*Fourth*, especially after disturbances, shrubs have greater bark area for sprouting and potential development of new organs compared to trees with the same above-ground woody volume. Epicormic and dormant adventitious buds underneath the bark of stems, stem bases, and branches can contribute to growth. Assuming that a small tree and a small shrub have equal density of such buds on their stems above ground, a shrub with its greater bark area may gain a growth advantage by higher sprouting potential. Sprouts will also have more space available for growth on the spread-out stems of a shrub than if they all grow on a single-stem tree. However, as both external conditions (e.g., light, water availability, disturbances) and internal conditions (e.g., growth hormones and stored resources) may trigger sprouting, the role of bark area *per se* remains to be clarified.

*Fifth*, assuming the same branching pattern (*a*_*s*_ = *a*_*t*_), a small shrub can produce more branches and twigs than a small tree (Figure 3D) and its canopy can expand horizontally to capture more light than a small tree that tends to grow mainly upwards (see also Pickett and Kempf, 1980; Givnish, 1984; Küppers, 1989; Sun et al., 2010).

*Sixth*, multiple stems in a shrub may allow continued horizontal growth. The stems can grow close to the ground; as their length and mass increase, some stems lean against the ground allowing new roots and vertical shoots to develop, further horizontally expanding the canopy (one example in Figure 2B). Pickett and Kempf (1980) suggested that “shrubs represent a horizontally oriented strategy for reduction of [self-]shading,” referring to clones and root suckers in the shrubs they studied (see Discussion).

Based on the six traits and the mechanisms described above, we predicted higher above-ground growth rate in small shrubs than in small trees with the same woody volume, and tested this prediction by a review of the literature. Using the Web of Science database and reference lists in published articles and books, we searched for articles containing the term “shrub” where the authors had quantified growth in both shrubs and trees. After exhaustive search, we found 14 such studies. We also looked for evidence for Hypotheses 2–4, but not systematically.

We categorized the 14 studies into those with (1) disturbed habitat and mainly resprouting plants—five studies, (2) disturbed habitat and mainly seeders (pioneer plants)—one study, (3) laboratory experiments—three studies, (4) field experiments—two studies, and (5) natural colonization and growth—one study. Two studies were not easy to categorize. The 14 studies are presented in Table 2. Studies of both absolute and relative growth rate were included. Overall, we find support for our prediction in 12 studies (good support in three studies), while two studies were inconclusive (see Table 2). For a description of the 14 studies with additional information, see Data Sheet 2 in Supplementary Material.

**Table 2:** **Result of literature review to test the prediction from Hypothesis 1: higher above-ground growth rate in small shrubs than in small trees**.

**Category and country**	**Habitat**	**Comparison**	**Main result**	**Type of evidence for Hypothesis 1 (++, +, +/−, −)**	**References**
**DISTURBED HABITAT, MAINLY RESPROUTING**					
Brazil	Grassland	2 shrub and 3 tree species	Shrubs regained more basal area and height than trees after fire/cutting	++	Hermann et al., 2012
Brazil	Forest-grassland ecotone	38 shrub and 42 larger woody species	Shorter multi-stemmed shrubs dominated early regrowth after fire	+	Müller et al., 2007
Brazil	Savanna (Cerrado)	4 shrubs/subshrubs and 3 tree species	Diameter growth^a^; basal area, biomass, and heights not given	+/−	Hoffmann and Solbrig, 2003
Sweden	Mixed forest with Quercus	1 shrub and 13 tree species	Shrubs had higher growth rate and survival rate than trees after partial cutting	+	Leonardsson and Götmark, 2015
Japan	Mixed forest with Quercus and Carpinus	7 shrub and 24 larger woody species	Shrubs had stronger resprouting than the other species	+	Shibata et al., 2014
**DISTURBED HABITAT, MAINLY SEEDERS**					
Mexico	Tropical deciduous forest	47 species; mix, but more trees than shrubs	Shrubs and trees did not differ in height growth after clear-cut and burn^b^	+/−	Miller and Kauffman, 1998
**LABORATORY EXPERIMENTS**					
Lab	Species from British isles and northern Spain	25 shrub/sub-shrub and 55 tree species	Shrubs had higher relative growth rate than trees (only tested up to day 21)	+	Cornelissen et al., 1996
Lab	The tropics; meta-analysis of 15 studies	17 shrub, 12 intermediate and 61 tree species	Shrubs accumulated more biomass than trees after nutrient addition (*P* < 0.07)^c^	+	Lawrence, 2003
Lab	Karst habitats, SW China	2 shrub and 4 tree species	Shrubs had higher biomass increase than “most of the trees”^d^	+	Liu et al., 2011
**FIELD EXPERIMENTS**					
Mexico	Tropical oak forest: open, edge, and interior habitat	2 shrub and 3 tree species (seedlings planted)	Shrubs had higher biomass growth, larger root systems, and higher survival than trees^d^	++	Asbjornsen et al., 2004
Spain, highlands	Forest, shrub-land and open	4 shrub^e^, and 4 tree species (seeds sown)	Shrubs tended to survive better than trees, especially under dry conditions^d^	+	Matias et al., 2012
**NATURAL COLONIZATION AND GROWTH**					
USA, New York state	Abandoned fields	2 shrub and 2 tree species	Shrubs emerged better per seed, survived better, grew better, and became taller than the trees	++	Gardescu and Marks, 2004
**OTHER STUDIES**					
Lab, and experiments^f^ (also herbs)	Diverse conditions	9 studies of shrubs, 27 studies of trees	Shrubs had higher median relative growth rate than trees	+	Houghton et al., 2013
Australia	Post-fire successional habitat	17 shrub-like, 2 taller tree-like species	Shrub-like outpaced tree-like species in height growth (early growth)	+	Falster and Westoby, 2005

a *Difficult to compare growth data in publication*.

b *Similar height growth of shrubs and trees generally implies higher (above-ground) biomass growth in the shrubs, since they have more stems*.

c *Bonferroni-test that may be considered conservative*.

d *Tested drought tolerance, or related the study to drought tolerance*.

e *Two species referred to as scrub and broom by authors are considered shrubs here*.

f *Controlled studies (laboratory and field experiments); studies of only trees, and of only shrubs, also included. Shrubs vs. trees not directly tested (their Figure 1)*.

*Types of evidence: ++, good support for prediction; +, support; +/−, inconclusive; −, contradicts the prediction*.

### Hypothesis 2: the fast maturity of shrubs enables earlier seed production compared to trees

Because a shrub does not grow tall, it will reach reproductive size earlier (e.g., Hoffmann and Solbrig, 2003), and produce seeds earlier than a tree (e.g., Hermann et al., 2012). This gives shrubs an extra fitness benefit after processes that reduce tree dominance. Early seed production should facilitate seed dispersal to new, unoccupied patches. A tall tree with a large canopy can produce many more seeds than a shrub, but for most trees, the time lag in seed set is a disadvantage. This hypothesis may be less relevant for tropical rain forests where small tree species also occur (e.g., understory treelets), with apparently fewer multi-stemmed shrubs.

### Hypothesis 3: the multiple stems in shrubs insure future survival and growth if one or more stems die

A single-stemmed tree faces a lethal risk if the stem breaks and dies due to e.g., harsh weather conditions, falling trees/branches, drought, disease, or browsing, and trampling animals. In contrast, a shrub can afford to lose some of its stems and still survive (Wilson, 1995; Sheffer et al., 2014). Loss of a stem during the growth season will result in loss of foliage, reducing C uptake. A tree would lose its entire canopy, whereas a shrub would only lose part of it. In the dormant season, if a single-stemmed tree breaks it loses all its dormant terminal and lateral buds, delaying foliage, and canopy development and C uptake the following year. Further, woody plants may store resources (e.g., nitrogen, water, and non-structural carbohydrates) within the stems for re-growth, and a tree loses all such stored resources if its stem breaks near the ground. Multi-stemmed shrubs may also have an advantage over single-stemmed trees if a stem suffers hydraulic failure. This may be especially true for hydraulically modular shrubs, such as *Ambrosia dumosa* in arid ecosystems in California, USA (Espino and Schenk, 2009).

### Hypothesis 4: the short stems of shrubs improve survival through three traits, compared to tall tree stems

We suggest that short shrubs have at least three structural traits that improve survival, compared to taller trees. Being short or tall is a trade-off; trees gain other advantages from being tall, as mentioned at the end of Section The Occurrence and Ecological Importance of Shrubs.

*First*, shrubs can bend and thus survive storms, snow load, avalanches, etc., which may otherwise result in stem breakage. In cold and alpine areas, low vegetation survives extreme weather and strong winds better, due to better aerodynamic resistance and improved temperature conditions (Grace, 1988; Hallinger et al., 2010; Neuner, 2014). In areas with landslides and avalanches, shrubs are favored by short and flexible stems compared to trees, which may fall (review in Givnish, 1995; Stokes et al., 2012). Recently, Larjavaara (2015) emphasized the advantages of stem flexibility in shrubs, and suggested that this limits their height.

*Second*, shrubs can have a wide canopy with leaning stems to capture more light, since low height reduces the cost of the bending moment of leaning stems. Bending moment increases with increasing stem length and stem angle (Figure 4), so that the cost is much higher for a tree with leaning stems. We neglect branches for simplicity, so the actual bending moment of a stem with its canopy would be greater than in Figure 4, and it would also be increased by snow and wind (e.g., Spatz and Bruechert, 2000). Falster and Westoby (2005) commented that “Multiple stems are thought to limit maximum height since they emerge at an angle and are less securely attached to the roots” (see also Kruger et al., 1997).

*Third*, the lower height of shrubs compared to trees should reduce the risk of cavitation due to drought and freezing. The maximum height of trees is partly determined by the problem of getting water to tall canopies. Water shortage can cause embolism in the xylem (Tyree and Sperry, 1989), and the risk of cavitation increases with stem height because of gravity (Ryan and Yoder, 1997). Freeze-thaw cycles during winter can cause similar problems (Tyree and Sperry, 1989; Zhu et al., 2000). This may be common in alpine and arctic habitats, where trees are disfavored especially in windy conditions, when temperatures drop. Because shrubs are shorter, they are less likely than trees to suffer from these problems, which occur in many habitat types. In addition, snow can more easily cover a shrub than a tree and protect it against low temperatures.

A possible further component of Hypothesis 4 might be: since shrubs are generally lower than trees, they may invest relatively less in support structure, and can invest more in e.g., foliage and roots, as suggested by Whittaker and Woodwell (1968). However, an overview and analysis of this suggestion is needed, including a definition of “support structure.” One would need to compare small trees and shrubs, as well as large trees and shrubs.

## Discussion

We found support for our basic model, Hypothesis 1, and the prediction of higher growth rate in small shrubs than in small trees. Since shrubs occur in several biomes and many habitats and include numerous species, multiple hypotheses are needed to fully clarify their adaptive significance. Shrubs exhibit striking morphological diversity and adaptations within regions and across gradients (e.g., Schenk et al., 2008). Many studies relate shrubs and multi-stemmed trees to disturbed and low-productive habitats (e.g., Rundel, 1991; Wilson, 1995; Hoffmann and Moreira, 2002; Bellingham and Sparrow, 2009). Our Hypotheses 1 and 2 are independent of habitat, while the advantages predicted by Hypotheses 3 and 4 depend on disturbances, morphology, extreme weather, and climate. Shrubs seem to survive by combinations of fast growth and persistence (e.g., Kanno et al., 2001; Tanentzap et al., 2012), including early seed production and long-distance dispersal (e.g., by wind and birds).

An important result from our model, and the first functional trait under Hypothesis 1, was that shrubs have greater total sapwood area compared to single-stemmed trees of similar size. For an individual tree or shrub stem, the cross-sectional area of the sapwood is correlated with its total leaf area and mass (Waring et al., 1982; Meadows and Hodges, 2002; Wang, 2005; Rance et al., 2014; Issoufou et al., 2015) and thus with C uptake. Most studies focus on trees, and for shrubs we found only a handful of studies relating sapwood area to leaf area/mass, or the Huber value (HV, conductive xylem per leaf area) (Gartner, 1991; Wang, 2005; Issoufou et al., 2015). Wang (2005) reported data for shrubs and trees within the same site; he studied hydraulic conductivity and the ratio of sapwood cross-sectional area and leaf area (HV) in 10 trees and four shrubs in Canada and reported similar values in trees and shrubs. His study supports our assumption of a similar ratio of sapwood cross-sectional area and leaf area in trees and shrubs, giving shrubs growth advantages over trees of similar size (i.e., of the same above-ground woody volume, as in our model).

One mechanism in Hypothesis 1 was that shrubs should sprout better than small trees from buds because of their larger surface area and widespread stems with more space for sprouts. Sprouting is involved in the fast growth of shrubs (Table 2) but we found no study that directly compared bud density or bud numbers and the initiation of sprouts on shrubs and small trees. Bond and van Wilgen (1996) distinguished basal and crown sprouting in woody plants in response to fire. The positions of buds and sprouts on woody plants are rarely described in published studies. In South African savanna after fire, sprouting near the ground dominated for shrubs (A. Zizka, pers. com.), presumably because fire kills buds higher up (see also Bond and van Wilgen, 1996; Hoffmann and Solbrig, 2003). Sprouting varies much among shrub species and with height growth (Bond and Midgley, 2001); some shrubs also sprout along stems (Figure 2B, and e.g., Lunt, 2011).

Shrubs may also flower and set seeds earlier than trees (Hypothesis 2); although this seems likely (e.g., Hoffmann and Solbrig, 2003; Hermann et al., 2012), a review and more empirical studies are desirable. Hypothesis 3 predicts that shrubs should survive stem breakage better than trees, for which there is little evidence (but see Shibata et al., 2014; Leonardsson and Götmark, 2015). In general, Hypotheses 3 and 4 would predict lower mortality in shrubs than trees. Condit et al. (1995) studied the mortality of tropical trees and shrubs during and after a drought on Barro Colorado Island (BCI), Panama; shrubs had higher overall mortality rates than trees, but shrubs had mortality rates less affected by the drought than trees. Lopez et al. (2005) analyzed xylem vulnerability to cavitation in five tree species and four shrub species on BCI (all shrubs were shade-tolerant). The four shrubs were on average less vulnerable to cavitation than the trees (Lopez et al., 2005; Table 2), and a shrub was the least vulnerable.

One contributing factor for fast growth in shrubs could be lower investment in wood, i.e., lower xylem density. If there is less need for structural strength in short shrub stems, they can invest more in growth. Castro-Diez et al. (1998) found that “shrub seedlings had less dense stem tissues than tree seedlings,” and added “possibly because they need less investment in long-term strength and stature.” However, two groups of 65 shrub species and 135 tree species from Argentina, Mexico, and the US did not differ in wood density (Martínez-Cabrera et al., 2011). Moreover, in a recent study of three co-occurring woody species that differed in maximum height (McCulloh et al., 2015), the shortest (a *Corylus* shrub) had the highest stem wood density, and the tallest (an *Alnus* tree) had lowest wood density (stems compared at similar plant heights).

Martínez-Cabrera et al. (2011) reported that shrubs had lower vessel diameter, and a higher density of vessels than trees. This was in addition reported for alpine shrubs compared to trees (Noshiro and Suzuki, 2001), and such vessels may reduce the risk of embolism—see also Lopez et al. (2005). McCulloh et al. (2015, see previous paragraph) suggested that vulnerability to embolism increases with maximum potential height in the species. A reviewer suggested that plants “might be short (and shrubby) because they have inefficient, short, narrow vessels…which alone would make them less vulnerable to cavitation,” but it is also possible that both vessel traits and low height reduce the risk of embolism in shrubs. Worth noting is that Maherali et al. (2004), using data from a global database, found a large overlap in cavitation risk between major groups, life- and growth-forms, where shrubs on average were less vulnerable to cavitation than trees.

After severe damage or sudden increased light availability, a small tree or tree stump can sprout and change into shrub growth form. We suggest that our hypotheses contribute to explaining this response in trees, and thus may form parts of a theory of multi-stemming in woody plants. In trees, a change to a multi-stemmed growth form may be an attempt to survive a difficult situation and may sometimes fail and increase mortality, depending on tree size, species, and conditions (e.g., Del Tredici, 2001; Leonardsson and Götmark, 2015). One study of single- and multi-stemmed large *Pinus strobus* is consistent with our Hypothesis 1, since the latter, of a given age, grew better (higher volume production) than individuals with a single stem (Chamberlin and Aarssen, 1996). In addition, based on long-term data, Bellingham and Sparrow (2009) reported lower mortality (and lower recruitment) in multi-stemmed than in single-stemmed trees in montane rain forest.

Hypothesis 4 posits that low height in shrubs allows the stems to spread wide, since the bending moment of short stems is low even if they lean outward. The same bending moment calculations (see Figure 4) may also help explain why tall trees usually have a single stem and why multi-stemmed trees tend not to have the same shape as shrubs. A tall multi-stemmed tree with wide-spread leaning stems would need to invest much in stem strength (including reaction wood, Du and Yamamoto, 2007) to counteract the large bending moment; for very tall trees this would even likely be impossible. Such trees are therefore rare. Instead, a multi-stemmed tree might seek to lessen the bending moment with upward-tending stems (see “uprising stem” in Figures 2C,D, 4). But such stems would be close to each other and would each have a canopy smaller than that of an isolated stem, leading to an increased proportion of support structure for the tree as a whole. The tree would therefore not gain much in C uptake by having multiple stems. This may help to define and distinguish trees (single- and multi-stemmed) from shrubs, and help explain why shrubs do not evolve to “trubs,” intermediate in size between trees and shrubs (Sheffer et al., 2014). More detailed models, with empirical tests, are needed to clarify the growth form of stems in multi-stemmed trees.

Shrubs are often subjected to browsing. Zizka et al. (2014) suggested that the dense growth form of shrubs could protect the inner crown parts and their foliage from herbivory (a dilution effect). In the understory of temperate forest, deer grazing had little effect on growth and stem survival in the shrubs *Corylus avellana* and *Crataegus* sp. (Tanentzap et al., 2012). Livestock and grazing may also spread shrubs (Naito and Cairns, 2011). Possibly, small trees may be more susceptible to browsing than shrubs.

To assess and improve our model and hypotheses, the root systems of small shrubs and small trees are of considerable interest. Shrub roots can grow deep into the ground (Jackson et al., 1996; Schenk and Jackson, 2002). Whittaker and Woodwell (1968) suggested that shrubs, with smaller expenditure on supporting stem and branch tissue than trees, can allocate “a larger fraction of production [to] root growth,” to “survive fire, browsing, and drought.” A large tree must invest in structurally strong roots to support its size, while a shrub might develop relatively finer roots. But it is unclear whether small trees invest much in roots, and if they do so when they compete with shrubs. If a shrub can grow fast, it will be able to invest much in roots. In Asbjornsen's et al. (2004) study, the two shrubs grew better, and produced longer and more branched roots than the three trees. In Whittaker and Woodwell's (1968) study, the mean root/shoot ratio was clearly higher for four shrub species than for three tree species (p. 7), but in contrast to Asbjornsen et al. (2004), they report values for large trees (exact sizes not given) and for (small) shrubs.

Clonal growth is more common among trees and shrubs in “very harsh, resource-poor, or highly disturbed habitats” (Peterson and Jones, 1997). Some authors regard clumped stems of a single shrub individual as a clone, but it is important to distinguish clonal shrubs that grow by root suckers or runners and form new distant stems or stem clumps. To judge from the literature, it is unclear whether shrubs are clonal in this way more often than trees. Shrubs, compared to trees, may more often be clonal through layering, where nodes of lying stems root and sprout (one component in Hypothesis 1).

Finally, a comprehensive review of the adaptive significance of the shrub growth form seems non-existent (or is hard to find). Trees and herbs dominate the scientific literature: a search in the Web of Science for “tree^*^ AND leaf^*^” gave 40,324 publications (18 March, 2016), and for “shrub^*^ AND leaf^*^” 5658 publications (“tree^*^ AND shrub^*^ AND leaf^*^”: 1839). Classical and much-cited publications on woody plants usually exclude shrubs, or focus strongly on trees (e.g., Horn, 1971; Connell, 1978; Loehle, 2000; Petit and Hampe, 2006; Thomas, 2014; but see Bond and van Wilgen, 1996 and recent studies of shrubland and climate change). The literature on woody plants in fire-prone ecosystems includes studies of shrubs, though with focus on fire or sprouting (e.g., Bond and Midgley, 2001).

Why is the shrub growth form neglected? Partly perhaps because it is difficult to define shrubs precisely or because of their often low direct economic value, but aesthetical aspects are probably also involved: many of the shrub-covered areas do not attract people. Negative words are common, such as “scrubby,” “thicket,” “encroachment,” “broussaille” (French), “sly” (Swedish); even “shrub” may sound negative. Many shrubs reduce the view of the surroundings, and good view is important for humans that hunt prey or seek charismatic species (Orians, 1986; Gray and Bond, 2013). Raunkiaer (1934) did not classify shrubs separately, but listed five other categories in the “most widely used system” of plant life forms (Archibold, 1995, p. 2) which may have led botanists to overlook shrubs. In contrast, shrubs are popular in horticulture, and the interest in shrubs increases.

## Conclusions and future studies

We have attempted to clarify why shrubs are successful in many habitats, including those where trees grow. Hypothesis 1 predicts that small shrubs should have higher growth rates than small trees, and we find evidence for that. Shrubs, compared to trees, should have earlier seed set and dispersal (Hypothesis 2), should have higher survival in extreme conditions and weather, and can benefit from having several stems and low height (Hypotheses 3 and 4). Although all hypotheses have some support, more studies and more detailed models are needed, including laboratory and field experiments where shrubs and trees are sown or planted in different habitat types and followed at least until the shrubs are fully grown. Growth rates, morphological traits, and ecophysiology should be analyzed in detail, and parts of the plant populations should be harvested at two or more stages to analyze whole plants.

Trees are more successful than shrubs in many areas under certain climatic conditions, where tall trees can dominate or control shrubs. The large distributions of some shrub-dominated communities may partly be due to pre-historical and historical human overexploitation of such areas, and of trees (Williams, 2006). Examples include Iceland (Diamond, 2005) and the West European heathlands dominated by the shrub *Calluna vulgaris* (Vandvik et al., 2014, and references therein). A review of the historical role of humans in the distribution of shrublands globally would be valuable.

Currently, our results are of interest for studies of shrubs expanding into savannas, rangelands, and grasslands, and for studies related to climate change, C pools, and habitat management (see e.g., Knapp et al., 2008; Naito and Cairns, 2011; Ratajczak et al., 2012; Gray and Bond, 2013; Conti et al., 2014; Ogden, 2015). Studies on “shrubification” in such open habitats (Naito and Cairns, 2011; Formica et al., 2014) can be reviewed: did the study areas lack trees, or did trees occur there but did not increase? A related, much studied and old theme is that shrubs may facilitate tree regeneration by providing protection for small tree plants (e.g., Jefferies, 1885; Filazzola and Lortie, 2014). Researchers in this field should also ask: what made the shrubs more successful than the trees initially? Moreover, some genera contain both trees and shrubs, e.g., *Salix, Quercus, Camellia, Acacia*, and *Juniperus*. The species may be closely related, or one species may be highly variable, existing both as shrub and tree. A review of such species, their occurrence, and existing studies of them is of interest, and may clarify selection pressures acting on species and growth forms.

## Author contributions

FG conceived the topic and problem, and the initial approach. EG constructed the models and ran the calculations. FG, EG, and AJ developed hypotheses, partly from a review of the literature (made by FG, to some extent also EG and AJ). FG, EG, and AJ wrote the manuscript together.

### Conflict of interest statement

The authors declare that the research was conducted in the absence of any commercial or financial relationships that could be construed as a potential conflict of interest.
