# Supplementary material for: Why Be a Shrub? A Basic Model and Hypotheses for the Adaptive Values of a Common Growth Form
Source: Front Plant Sci. 2016 Jul 26;7:1095. doi: 10.3389/fpls.2016.01095 (PMC4961008; doi:10.3389/fpls.2016.01095)
Supplement: Supplementary file 2 [file DataSheet2.docx]

**Appendix 1** Presentation of 14 studies used to test prediction from Hypothesis 1: higher above-ground growth rate in a small shrub than in a small tree (see also Table 2)

________________________________________________________________________

*Disturbed habitat, mainly resprouting*:

In two Brazilian grassland sites, subjected to fire or cutting, Hermann et al. (2012) studied small individuals of two forest tree species, and two shrub species. After one year, the shrubs regained 73-142% of pre-disturbance basal area, as opposed to 14-24% in trees; as for height above ground, shrubs regained 73% and trees 22-46% of the pre-disturbance level (they omitted a “re-seeder” shrub that did not sprout). The post-disturbance mean height per species (tallest shoot) was 48 cm for shrubs (n=3) and 46 cm (n=4) for trees (Hermann et al., 2012: Table 3). In a second study in southern Brazil, Müller et al. (2007) studied a forest-grassland ecotone and woody plant functional traits in 80 species from 40 families in relation to fire (partly by experimental burning). For grassland, “single-stemmed shrubs predominated in late post-fire recovery (3–4 years), while shorter multi-stemmed shrubs [predominated] in recently burned areas (3 months to 1 year)”. Forest trees occurred in border plots, or as (taller) adults. In Brazilian regularly burned savanna (cerrado), topkill of plants due to fire was common, and regrowth was studied (stem diameters; Hoffmann and Solbrig 2003). Sub-shrubs and shrubs had resprouted to reproductive size after one year (in three of four species) whereas the trees (three species) then were far from reproductive size (no height data presented, and shrubs mature at smaller sizes). Unburned shrubs and trees did not seem to differ in diameter growth, but note that equal diameter growth for shrubs and trees implies higher (above-ground) biomass growth for shrubs, since they have more stems.

In temperate forest in Sweden subjected to experimental thinning, the survival and growth of cut stumps in 13 tree species and one shrub were studied for a 9-year period (Leonardsson and Götmark, 2015). The shrub *Corylus avellana* had much higher survival and growth than all trees. In Japan, Shibata et al. (2014) studied growth of stumps on clear-cuts. Among 7 shrubs and 24 larger woody species/trees, shrubs had stronger resprouting and ”retained the ability to resprout throughout their lifetimes”.

*Disturbed habitat, mainly seeders*:

In clear-cut and slash-burned tropical deciduous forest in Mexico (Miller and Kauffman, 1998), no difference in the height of shrubs and trees was recorded after the disturbance (plants not studied before disturbance). The dominating shrubs were pioneer species. Note that similar height growth of shrubs and trees generally implies higher (above-ground) biomass growth in the shrubs, since they have more stems.

*Laboratory experiments*:

Cornelissen et al. (1996) planted seeds of 80 woody species from the British Isles and northern Spain in the lab, and examined the growth up to 21 days. Among deciduous genera (climbers/scramblers, shrubs, trees) shrubs had on average 17% higher relative growth rates than trees. In 33 evergreen species, procumbent subshrubs and shrubs all had higher mean relative growth rate than trees (though not significantly so). Note that such young shrubs probably do not have many stems yet, and thus do not get much growth advantage as predicted by Hypothesis 1. Lawrence (2003) reported a meta-analysis of studies of nutrient addition in 90 tropical species, and the ratio of high-nutrient response to control response. Shrubs responded more than trees in biomass accumulation (P=0.07 with conservative Bonferroni testing). In China, Liu et al. (2011) tested drought tolerance for two shrubs (one evergreen, one deciduous) and four trees (one evergreen, three deciduous) over 100 days of growth in 2-year-old plants. The two shrubs had higher water status, higher photosynthetic capacity, and larger biomass increase “than most of the trees”, but the deciduous trees grew better under favourable conditions.

*Field experiments*:

In a well-designed study in fragmented tropical oak forest in Mexico, seedlings of two shrub species (*Dodonea viscosa*, *Rhus virens*) and three tree species (two *Quercus*, one *Pinus*) were planted in the open, in edge, and in forested habitat, and harvested after two years (Asbjornsen et al., 2004). The shrubs had clearly lower mortality than the trees, had higher biomass growth rate, and larger and deeper root systems. The (small) shrubs apparently withstood drought better than the (small) trees. In Spanish highlands, Matias et al. (2012) tested drought tolerance experimentally for plants (sown seeds) in three habitats (forest, shrubland, and in the open) and harvested plants after two growth seasons. Four species were trees and four shrubs (one “scrub” and one “broom”) and they differed in shade tolerance. Survival and drought tolerance was consistently low in two trees (*Acer*, *Pinus*), relatively low also in *Sorbus*, but high in *Quercus ilex*. The shrubs generally succeeded better, except *Salvia* in forest and *Berberis* in the open.

*Natural colonization and growth*:

Gardescu and Marks (2004) studied seed input, plant emergence, and growth in two shrubs (*Cornus racemosa*, *Viburnum dentatum*) and two trees (*Fraxinus* *americana*, *Acer rubrum*) in abandoned fields in New York State, USA, over six years. Emergence per seed was much higher in shrubs. Seedlings of shrubs often survived better than did trees, and the shrubs grew better. For example, median heights of five-year-old plants were 15 cm (*Viburnum*) and 17 cm (*Cornus*), while trees then had heights of 10-12 cm. The maximum heights differed even more: 17-32 cm for the trees vs. 54-55 cm for the shrubs.

In addition, two studies could not be classified into the groups above. Houghton et al. (2013) compared relative growth rate in herbs and woody plants, using only controlled studies and early growth (lab or experimental studies). They separated trees and shrubs in their Figure 1, which shows that shrubs had higher median growth rates than trees (studies of only trees, of only shrubs, and of both forms in same study, included). Falster and Westoby (2005) studied height growth by chronosequences in 12 reseeders and 8 resprouters in post-fire successional habitat in Australia. Among reseeders (shrubs, and two taller species that also seem to occur as single-stemmed trees), the shrubs grew faster and above the two trees up to about year 11, when they were overtopped by them (see their Figure 3). The resprouters contained only one tree (included also as reseeder), overtopping these shrubs after about 2 years.

SUPPORTING INFORMATION

Additional Supporting Information may be found in the online version of this article at the publisher’s web-site. Contains the basic model with calculations implemented in Matlab. The files may also be opened in Word or other programs.
