# Supplementary material for: Why Be a Shrub? A Basic Model and Hypotheses for the Adaptive Values of a Common Growth Form
Source: Front Plant Sci. 2016 Jul 26;7:1095. doi: 10.3389/fpls.2016.01095 (PMC4961008; doi:10.3389/fpls.2016.01095)
Supplement: Supplementary file 1 [file DataSheet1.ZIP › supplementary material/_README.doc]

The main file is trees_and_shrubs.m, which contains code to generate the figures in the article (as well as other figures which do not appear in the article). The main file calls on all the other files, which are functions to calculate tree volume, shrub volume, etc. Most should have self-explanatory names and also contain comments. The file parameters_trees_shrubs.dat contains all parameters, which are loaded in the beginning of all files. The main file contains explanations of the parameters.

The files rad.m as well as all files ending in "n" (such as treevolume_n.m) use a different model for the relationship between basal radius and length for stems and branches. In treevolume.m etc, the relationship is assumed to be proportional, and in treevolume_n.m a relationship from Niklas & Spatz (2004) is used instead (see main article). The files for bending moment also use rad.m.

Note that Matlab files can be opened and read in Word, if you do not use Matlab (though of course the code cannot be run there).

There are also three supplementary graphs which do not appear in the article.
